# Association Between Physical Activity and Serum 25-Hydroxyvitamin D Levels Among Adolescents in Northern Sudan: A School-Based Cross-Sectional Study

**DOI:** 10.3390/nu18060986

**Published:** 2026-03-20

**Authors:** Ahmed A. Hassan, Mustafa I. Elbashir, Abdullah Al-Nafeesah, Ashwaq AlEed, Ishag Adam

**Affiliations:** 1 Faculty of Medicine, University of Khartoum, Khartoum 11115, Sudan; 2 Department of Pediatrics, College of Medicine, Qassim University, Buraydah 51452, Saudi Arabia; a.alnafeesah@qu.edu.sa; 3 Department of Obstetrics and Gynecology, College of Medicine, Qassim University, Buraidah 52571, Saudi Arabia

**Keywords:** adolescent, age, vitamin D, 25(OH)D, physical activity, female, education

## Abstract

**Background:** The association between physical activity and vitamin D status is not yet fully understood. This study aims to investigate the prevalence of physical inactivity and its associated factors, including serum 25-hydroxyvitamin D (25[OH]D) concentration, among adolescents in Northern Sudan. **Methods:** A school-based cross-sectional study was conducted in Almatamah, River Nile State, Sudan, and a questionnaire was used to collect sociodemographic data. Standardized methods were used to measure physical activity and serum 25(OH)D levels. Physical activity was expressed as metabolic equivalent minutes per week (MET-min/week). A multivariate binary regression was performed. **Results:** Three hundred and thirteen adolescents [159 (50.8%) males and 154 (49.2%) females] were enrolled in the study. The median (interquartile, IQR) values for age, 25(OH)D, and physical activity were 15.1 (14.0–16.2) years, 20.2 (9.6–31.2) ng/mL, and 1080 (495–3360) MET-min/week, respectively. The median (IQR) physical activity score was higher in males than in females [3287.5 (1680.0–4659.0) MET-min/week vs. 495.0 (314.3–990.0) MET-min/week]. Of the enrolled adolescents, 220 (70.3%) had inadequate physical activity levels (<3000 MET-min/week). Serum 25(OH)D level was significantly lower in adolescents with inadequate physical activity than in those with adequate physical activity levels [17.7 (7.8–28.0) ng/mL vs. 26.4 (17.3–36.8) ng/mL]. In the multivariable binary analysis, female sex (adjusted odds ratio [AOR]: 35.0; 95% CI: 13.89–88.08), a lower paternal education level (AOR: 2.812; 95% CI: 1.39–5.70), and having a skilled father (AOR: 2.08; 95% CI: 1.05–4.12) were factors associated with inadequate physical activity among adolescents, whereas 25(OH)D levels were inversely associated with insufficient physical activity (AOR: 0.97; 95% CI: 0.95–0.99). **Conclusions:** Interventions are needed to address the high level of physical inactivity among adolescents in Northern Sudan, particularly among girls. Programs that promote physical activity both at home and school help ensure that children and adolescents maintain adequate physical activity and 25(OH)D levels.

## 1. Introduction

There is a global pandemic of physical inactivity among all age groups, including adolescents [1,2]. The prevalence of physical inactivity is high worldwide, with 84% of students classed as physically inactive and 37% classed as sedentary [3]. This is a significant concern, considering the association between physical inactivity and various health issues, including non-communicable diseases such as cardiovascular disease, type 2 diabetes mellitus, obesity, and mental health disorders [4,5,6]. According to the World Health Organization (WHO), adequate physical activity provides several physical and mental health benefits for adolescents: it improves physical fitness and cardiometabolic health, promotes bone health, encourages the healthy growth and development of muscles, reduces body fat, and improves motor and cognitive development [1]. The WHO has identified adolescence as a critical period for tackling disengagement from physical activity, making it important to develop strategies that target this age group [6]. With regard to community groups, adolescents, especially those in resource-limited settings, are vulnerable to physical inactivity and micronutrient deficiencies, including vitamin D, for reasons that include the high percentage of adolescents in the community (i.e., accounting for more than 20% of the Sudanese population) [7], a healthcare system ill-equipped to face increasing demand [8], poverty [9,10], conflicts, war, and psychiatric distress [11,12].

The prevalence of adequate physical activity has been reported as high in various countries, with rates increasing from countries with a low human development index, such as Sudan (8.8%), to those with a high index, such as Finland (58.7%) [2]. Various factors, such as decreasing age [13,14], being female [9,11,15,16], body mass index (BMI) [17], and maternal education [10], have been identified as potential risk factors for physical inactivity among adolescents. More attention has been paid to the association between physical activity and vitamin D status, particularly among adolescents [13,17,18,19,20,21,22,23,24]. In addition to sunlight exposure, diet and eating habits play a role in maintaining adequate vitamin D levels and proper calcium metabolism [25]. Vitamin D can be obtained through dietary sources—mainly fatty fish, liver, egg yolk, and fortified foods—and its bioavailability depends not only on intake but also on factors affecting absorption, such as the presence of dietary fat [26]. Likewise, calcium metabolism is influenced not only by physical activity and sun exposure but also by adequate dietary calcium intake, protein consumption, and other micronutrients such as magnesium and phosphorus [27]. Inadequate dietary patterns, restrictive diets, or the low consumption of foods rich in these nutrients may compromise calcium–vitamin D homeostasis, thereby affecting bone mineralization and growth during adolescence. Therefore, any analysis of the relationship between physical activity, vitamin D, and bone health should also incorporate these factors [25].

While some studies have reported a significant association between adolescents’ physical activity and 25-hydroxyvitamin D (25[OH]D) levels [13,17,18,19,20,21,22], other studies have found no such association [23,24]. For example, Al-Othman et al. [19] and Almehmadi et al. [20] observed that high physical activity levels were associated with high 25[OH]D levels, while Kim et al. [18] and Constantini et al. showed that reduced participation in physical activity was correlated with vitamin D deficiency [21]. Furthermore, Dong et al. found a positive association between physical activity and bone mineral density in vitamin D-deficient female adolescents. These findings suggest that physical activity mitigates vitamin D deficiency in adolescents [22].

The inconsistencies in data regarding the association between physical activity and 25[OH]D levels necessitate further study, especially in under-researched settings with limited resources, such as Sudan. A thorough understanding of the local context is needed to improve adolescents’ health. To achieve this, the status of physical activity among adolescents should be assessed and the associated factors, including 25(OH)D levels, should be investigated. The relevant parties can implement appropriate preventive measures to improve adolescents’ physical activity and vitamin D levels.

There is limited, inconsistent data available on physical inactivity among Sudanese adolescents, revealing a concerning trend [2,28,29,30] and underscoring the urgent need for interventions that promote physical activity among Sudanese adolescents.

Although issues with physical inactivity [28,29] and vitamin D deficiency [31,32] have been documented in Sudan, including among children and adolescents, no data have been published on the association between physical activity and vitamin D status among Sudanese adolescents. Notably, low awareness about physical activity [31] and vitamin D [32,33] has been documented, even among medical students. Such associations among adolescents need to be investigated to address both physical inactivity and 25(OH)D levels. Therefore, this study was conducted to investigate the prevalence of physical inactivity and its associated factors, including the associations of serum 25[OH]D concentration, among adolescents in Northern Sudan.

## 2. Materials and Methods

### 2.1. Study Design and Setting

This school-based cross-sectional study was conducted at governmental schools in Almatamah, Northern Sudan, from August to September 2022 and involved 313 adolescents. Almatamah is located in the River Nile State, approximately 140 km from Khartoum City, the capital of Sudan. The Wad Hamid district in Almatamah was selected for this study because it is an under-researched area and our previous work highlighted some concerns about adolescents’ health (i.e., vitamin D deficiency and poor academic performance) in this district [34]. The Strengthening the Reporting of Observational Studies in Epidemiology (STROBE) guidelines were strictly followed during this study [35].

### 2.2. Sampling Technique

The Almatamah locality consists of three districts, including the Wad Hamid district. There are 16 governmental schools (no private schools) for both boys and girls in Wad Hamid. Six schools were selected using simple random selection (i.e., the lottery method). A total of 5000 students were registered across the six schools, of whom 313 were selected (the desired sample size). The number of students recruited from each school depended on the total number of students at that school (i.e., probability proportional to size). Consequently, schools with larger student populations were overrepresented in the study sample. The sample was selected using a simple random technique (i.e., the lottery method) from a list of students at each school.

### 2.3. Inclusion and Exclusion Criteria

The sample included healthy adolescents aged 10–19 years. Students of younger (<10 years) or older (>19 years) ages were excluded, considering the WHO’s definition that “adolescence is the phase of life between childhood and adulthood, ages 10 to 19 years” [36]. Students who refused to participate in the study and those who were sick, pregnant, or lactating were also excluded.

### 2.4. Sample Size Calculation

As previously mentioned [37], and it has been reported that 59.5% of medical students in Khartoum, Sudan, were physically inactive, [31]. A sample size of 313 adolescents was estimated to be suitable for detecting a minimum difference (3.0 ng/mL) in the mean level of 25(OH)D between adolescents with and without inadequate physical activity. This sample size was calculated to detect a 5% difference at α = 0.05 with 80% power.

### 2.5. Study Variables and Measures

The questionnaire was adapted from previous studies [14,15,23,38] and included questions related to sociodemographic characteristics, such as age in years, gender (male or female), parents’ education levels (<secondary or ≥secondary), mother’s occupational status (housewife or employed), father’s occupational status (skilled worker or laborer), and adolescents’ smoking/snuff behaviors. The investigators trained six male and female medical officers for data collection.

The medical officers approached the selected students, after their guardians signed an informed consent form. They informed them that the study seeks their voluntary participation, that they have the right to withdraw at any time without providing reasons, and that preventive measures are in place to protect participants’ privacy, safety, and confidentiality, such as excluding personal identifiers during data collection. Weight, height, 25(OH)D, and physical activity were measured using standard procedures. Sociodemographic data, BMI, and 25(OH)D were considered secondary outcomes, whereas physical activity was the primary outcome.

### 2.6. Primary Outcome Measurements

The International Physical Activity Questionnaire (IPAQ) was applied to assess each adolescent’s physical activity status. The IPAQ has been reported to have a high validity and reliability [27,39]. A score for “Metabolic Equivalent (MET) minute/week” is obtained by multiplying minutes, days, and MET values (times of resting oxygen consumption). Moreover, the following categories are recommended for adolescents: physically inactive if the score is less than <600 MET-min/week; low physical activity if the score is between 600 and 3000 MET-min/week; and adequate physical activity if the score is more than >3000 MET-min/week [40]. The IPAQ has been widely used with adolescents in similar contexts [14,15].

### 2.7. Weight and Height Measurements

A standard procedure was used to measure the adolescents’ weight in kilograms (kg). The scales (a Seca 874 weighing scale, Seca, Hamburg, Germany) were well calibrated and set to zero before each measurement. Weight was measured to the nearest 100 g (g). The adolescents removed their shoes and any excess clothing and stood with their hands at their sides, minimizing movement. Height was measured to the nearest 0.1 cm (cm), with the adolescents standing straight against a wall, feet together. BMI was computed using the following equation: weight in kg divided by height in meters squared (kg/m^2^) [41].

### 2.8. Blood Sample Processing

Under septic conditions, 3 mL of blood from the cubital vein was collected from each adolescent and transferred into a plain tube. The blood samples were allowed to clot at room temperature. An enzyme-linked immunosorbent assay (450 nm, reference wavelength 620–650 nm) was performed to measure 25(OH)D according to the manufacturer’s instructions (Euroimmun, Lübeck, Germany). The manufacturer’s quality control measures and six standard solutions (calibrator) at levels between 0 and 120 ng/mL were applied to each assay. More information on this can be found in our previously published work [42]. In this study, serum 25(OH)D was analyzed as a continuous variable rather than a categorical one because different cutoff points are used to diagnose vitamin D deficiency [43,44].

### 2.9. Statistical Analysis

The collected data were entered into Statistical Product and Service Solutions (SPSS) for Windows (version 22.0; SPSS Inc., New York, NY, USA) for analysis. Continuous data, such as age, BMI, and physical activity scores, were evaluated for normality using the Kolmogorov–Smirnov test and were found to be non-normally distributed. They were therefore reported as median (interquartile range [IQR]) values. Categorical data were expressed in frequencies and percentages, and Spearman’s rank test was used to assess the correlation between 25(OH)D levels and physical activity scores. We performed a univariate analysis with physical inactivity as the dependent variable and 25(OH)D and sociodemographic variables (age, sex, BMI, parental education, and occupation) as independent variables. Following this, any variable with a *p*-value < 0.20 in the univariate analysis was entered into a multivariable logistic regression model to adjust for covariates. Odds ratios (ORs) and adjusted ORs with 95% confidence intervals (CIs) were calculated. A *p*-value < 0.05 was considered statistically significant.

## 3. Results

### 3.1. General Characteristics of the Adolescents

Of the 313 adolescents included in this study, 159 (50.8%) were boys and 154 (49.2%) were girls. The median (IQR) age and BMI values were 15.1 (14.0–16.2) years and 18.4 (16.4–21.5) kg/m^2^, respectively. The median (IQR) 25(OH)D was 20.2 (9.6–31.2) ng/mL. Regarding parents’ education levels, 189 (60.4%) mothers and 203 (64.9%) fathers had ≥secondary education. About one in 10—29 (9.3%)—of the adolescents’ mothers were employed, and roughly the same proportion of the adolescents—27 (8.6%)—were cigarette smokers or snuff users. The median (IQR) physical activity score was 1080 (495–3360) MET per week.

### 3.2. Factors Associated with Inadequate Physical Activity Among Adolescents

Of the 313 adolescents, 220 (70.3%) had inadequate physical activity levels (<3000 MET per week) and 93 (29.7%) had adequate physical activity levels (≥3000 MET per week). Adolescents with inadequate physical activity levels had low serum 25(OH)D (expressed as median [IQR] values) compared to those with adequate physical activity levels: 17.7 (7.8–28.0) ng/mL vs. 26.4 (17.3–36.8) ng/mL, *p* < 0.001 (Table 1). The median (IQR) physical activity score was higher for male adolescents than for females: 3287.5 (1680.0–4659.0) MET-min/week vs. 495.0 (314.3–990.0) MET-min/week, *p* < 0.001. There was a modest positive correlation (when the r/correlation coefficient was considered) between 25(OH)D concentrations and physical activity levels (correlation coefficient = 0.243, *p* < 0.001).

In the univariate logistic regression analysis, being female was associated with inadequate physical activity (unadjusted OR: 29.8; 95% CI: 12.45–71.42), while increasing 25(OH)D was less likely to be associated with inadequate physical activity (unadjusted OR: 0.96; 95% CI: 0.95–0.98); age, BMI, smoking/snuff behaviors, and parents’ education level and occupation status were not associated with physical activity (Table 1).

In the multivariable logistic regression analysis, being female (AOR: 35.0; 95% CI: 13.89–88.08), having a low level of paternal education (AOR: 2.812; 95% CI: 1.39–5.70), and having a skilled father (AOR: 2.08; 95% CI: 1.05–4.12) were associated with inadequate physical activity, and increasing 25(OH)D levels were less likely to be associated with inadequate physical activity (AOR: 0.97; 95% CI: 0.95–0.99; Table 2). OR = 0.97: This indicates a 3% decrease in the 1 ng/mL 25(OH)D levels in inadequate physical activity (small per unit). Because the change is very subtle (a decrease of only 0.03), 0.97 is very close to 1.0 (which means no effect). To calculate the results as the impact of a 10 ng/mL increase, we raised the OR (0.97) to the power of 10 (0.97^10^ ≈ 0.737). Thus, a 10 ng/mL increase in 25(OH)D is associated with a 1 − 0.73 = 0.27 or roughly a 26–30%. A goodness-of-fit test (Hosmer Lemeshow) in the logistic regression showed a good fit (*p* = 0.950). However, the wide 95% CI (13.89–88.08) for the association between females and inadequate physical activity indicated an inadequate sample size, as only six females had adequate physical activity (Table 1).

## 4. Discussion

Over two-thirds of the adolescents in this study were physically inactive. Females and adolescents whose fathers had low levels of education and were skilled workers were more likely to be physically inactive. In addition, increasing 25(OH)D levels were positively associated with physical activity among adolescents in Northern Sudan; that is, adolescents with increased 25(OH)D were less likely to have inadequate physical activity levels. This finding supports previous studies conducted in several countries—including Sudan—and global statistics indicating a high prevalence of physical inactivity among adolescents [2,28,29]. For example, the prevalence of inadequate physical activity was 70.3% in our study, comparable to the WHO estimate that 80% of adolescents worldwide do not meet the recommended physical activity levels [1]. In addition, our results align with previous studies that reported high levels of physical inactivity among adolescents, particularly among girls [2,28,29]. A study in Central Sudan involving 945 adolescents (507 male and 438 female) aged 14–18 years found that physical activity was infrequent (6.8%) and that sedentary behaviors, such as prolonged television viewing and internet use, were highly prevalent [29]. Another cross-sectional study in Central Sudan, which included 336 medical students aged 18 years or older, found that more than half (59.5%) of the students were physically inactive, with females (43.8%) more likely to be inactive [31]. A large study involving 325,219 students from 80 countries who participated in the Global School-based Student Health Survey between 2009 and 2018 reported that 84% were physically inactive [3].

The prevalence of inadequate physical activity (70.3%) among adolescents in our study was high compared to previously reported prevalence rates in countries such as Serbia (53.9%) [15] and Brazil (33.0%) [23].

This study revealed that female students were more likely to be physically inactive. This aligns with the results of several previous studies conducted in Khartoum, Sudan [28] and from North African and Middle Eastern countries [9,11,15,16]. In contrast, a previous study in Nepal [14] revealed no sex differences in physical activity. The lower prevalence of physical activity among adolescent females compared to males may be attributable to cultural factors; however, further research is needed. For example, this could be explained by movement restrictions imposed when children become adolescents (i.e., restrictions on access to common playgrounds). Aliss et al. reported that adolescent girls in Saudi Arabia spend more time watching television than adolescent boys [17]. In our results, the 95% CI (13.89–88.08) for the association between females and inadequate physical activity was wide, indicating an insufficient sample size, as only six females had adequate physical activity. This may be behind our results rather than a true association between female sex and inadequate physical activity.

This study showed that paternal factors, such as education level and occupational status, were more associated with physical activity than maternal ones. Adolescents with low-educated fathers were almost three times more likely to be physically inactive. Furthermore, adolescents with skilled fathers were more likely to be physically inactive. Associations involving paternal rather than maternal factors could be explained by paternal influences on family income and, consequently, on sedentary behavior. A study in Nepal revealed that adolescents from nuclear families, those with high family income levels, and individuals who used Wi-Fi had a high likelihood of internet addiction; they were more prone to engaging in inadequate physical activity than their counterparts who were not addicted to the internet [14]. A nationwide survey in Bangladesh found that higher paternal education levels and longer television viewing time were associated with inadequate physical activity [45]. A study of 12,770 adolescents aged 10–18 from developed countries, including Europe, Australia, Brazil, and the United States, found that higher maternal education was associated with lower objective physical activity and increased sedentary time [46]. In Sudan, the lack of influence of maternal factors, such as education and occupational status, on physical activity status could be attributed to the poor quality of education provided to girls—a consequence of their lower representation in the workforce [47] and their low contribution to a family’s socioeconomic status.

In the present study, increased 25(OH)D levels were positively associated with physical activity among adolescents. This association is supported by findings from previous studies conducted in different countries, including Arab countries [13,17,18,22]. In Saudi Arabia, a study that included 204 male adolescents aged 14–16 reported a significant correlation between physical activity scores and serum 25(OH)D levels (r = 0.418) [13]. Dong et al. studied 559 adolescents aged 14–18 and reported a significant positive association between 25(OH)D levels and vigorous physical activity and cardiovascular fitness [22]. In contrast, other studies have found no such association among adolescents [23,24]. The contradictory data between this study and studies conducted in Brazil [23] and South Korea [24] necessitate further research into the level and mechanisms by which 25(OH)D triggers physical activity and vice versa. For example, Constantini et al. emphasized the pivotal role of physical activity in bone health and highlighted its protective effect on preserving bone mass in the face of vitamin D deficiency [21]. The association between serum 25(OH)D levels and physical activity has implications for public health because imbalances between them could adversely affect metabolism, cardiovascular health, and mental health. It has been reported that higher physical activity may increase outdoor exposure and thus 25(OH)D [48].

The present study found no significant associations between age, BMI, cigarette smoking/snuff use, maternal education level, occupation status, and physical activity among adolescents. This is consistent with the results of previous studies that showed that physical activity is not associated with age [13,14], BMI [13,38], or cigarette smoking/snuff usage [4]. However, other studies have reported different results, namely that physical activity is associated with BMI [17] and maternal education [9].

The prevalence of physical activity and the factors associated with it in these studies may be due to differences in study setting (urban vs. rural) [16,49]. The current study focused on rural adolescents. Differences between the two settings may include varying exposure to sunlight (e.g., vitamin D), the availability of green spaces, and safety during physical activity, especially for females. The differences in physical activity prevalence and its associated factors should encourage researchers to study the epidemiology of physical inactivity in their countries and to develop specific, evidence-based solutions to promote physical activity among children and adolescents. This will, in turn, improve the status of global physical activity.

The present study’s results regarding the prevalence of physical activity among adolescents have public health implications for improving adolescents’ health. We identified modifiable factors associated with inadequate physical activity among adolescents, including vitamin D levels and their father’s education and employment status. This study can serve as a foundation for further research, especially with the ongoing war in Sudan, which has had severe negative impacts on population health and on the most vulnerable groups of children and adolescents [50]. This study also has implications for clinical practice, namely that adolescents with inadequate physical activity levels should be screened for vitamin D deficiency. Further high-quality research is required to confirm the association between vitamin D and physical activity on a large scale, considering the present study’s limitations.

The results of this study will be shared with policymakers and researchers in Sudan and elsewhere who are interested in adolescent health to address the lack of physical activity among children and adolescents and to encourage further studies. The ongoing war in Sudan is a primary challenge in implementing such recommendations, including conducting research. In Iraq, during the post-conflict period of 2017–2019, a cross-sectional study involving 600 adolescents aged 12–17 years showed that 472 (78%) were physically inactive [11]. Practicing physical activity not only exposes adolescents to sunlight but could also reduce the psychiatric distress and violent behaviors associated with vitamin D deficiency [12].

### Strengths and Limitations of the Study

The data obtained in this study add to the existing literature on physical activity among Sudanese adolescents [2,28,29], adults [31], and pregnant women [51]. However, the limitations of this study should be acknowledged to inform the design of future studies. First, the study’s cross-sectional design made it difficult to establish causality among the variables studied, particularly the direction of association between vitamin D and physical activity. Second, this study included adolescents from only one region of Sudan (Northern Sudan), which may limit the generalizability of its findings to the entire population. Third, no information on adolescents’ dietary patterns or those residing in urban areas was collected. These factors could influence physical activity and vitamin D status [5,29,49]. Moreover, calcium and other micronutrients such as magnesium and phosphorus were not assessed. Therefore, in future studies, any analysis of the relationship between physical activity, vitamin D, and bone health should also incorporate the assessment of dietary habits as a key and complementary determinant. Although the study’s univariate analysis is clearly presented, there may be confounders not included in the multivariate analysis, even if their results were not statistically significant or their *p*-values were greater than 0.20.

## 5. Conclusions

The high prevalence of physical inactivity among adolescents in Northern Sudan necessitates urgent interventions, particularly among females. Programs that promote physical activity at home and at school are essential for ensuring that children and adolescents maintain adequate physical activity and vitamin D levels. Further research is needed to consider the undeniable effects of diet and nutrition on calcium metabolism.

## Figures and Tables

**Table 1:** The characteristics of the studied adolescents and univariate analysis of factors associated with inadequate physical activity in Northern Sudan (n = 313).

Variable		Total (Number = 313)	Adolescents with Inadequate Physical Activity (<3000 Metabolic Equivalent Minute per Week (MET-min/week) (n = 220)	Adolescents with Adequate Physical Activity (≥3000 MET-min/week) (n = 93)	Unadjusted Odds Ratio (95% Confidence Interval)	*p*-Value
		Median (Interquartile range)				
Age, years		15.1 (14.0–16.2)	15.2 (14.0–16.4)	15.0 (13.9–15.7)	1.07 (0.92–1.26)	0.374
Body mass index, kg/m^2^		18.4 (16.4–21.5)	18.6 (16.7–21.6)	17.6 (16.0–21.5)	1.04 (0.96–1.11)	0.296
Serum 25-hydroxyvitamin D concentrations, ng/mL		20.2 (9.6–31.2)	17.7 (7.8–28.0)	26.4 (17.3–36.8)	0.96 (0.95–0.98)	<0.001
		Frequency (percentage)				
Sex	Male	159 (50.8)	72 (32.7)	87 (93.5)	Reference	
	Female	154 (49.2)	148 (67.3)	6 (6.5)	29.8 (12.45–71.42)	<0.001
Mother’s education level	≥secondary	189 (60.4)	134 (60.9)	55 (59.1)	Reference	
	<secondary	124 (39.6)	86 (39.1)	38 (40.9)	0.93 (0.57–1.52)	0.770
Father’s education level	≥secondary	203 (64.9)	137 (62.3)	66 (71.0)	Reference	
	<secondary	110 (35.0)	83 (37.7)	27 (29.0)	1.45 (0.88–2.50)	0.142
Mother’s occupation	Housewife	284 (90.7)	196 (89.1)	88 (94.6)	Reference	
	Employed	29 (9.3)	24 (10.9)	5 (5.4)	2.16 (0.80–5.83)	0.131
Father’s occupation	Laborer	188 (60.1)	126 (57.3)	62 (66.7)	Reference	
	Skilled worker	125 (39.9)	94 (42.7)	31 (33.3)	1.56 (0.91–2.68)	0.104
Cigarette smoking/snuff	No	286 (91.4)	204 (92.7)	82 (88.2)	Reference	
	Yes	27 (8.6)	16 (7.3)	11 (11.8)	0.59 (0.26–1.31)	0.194

**Table 2:** Multivariable logistic regression analysis of factors associated with inadequate physical activity among adolescents in Northern Sudan (n = 313).

Variable		Adjusted Odds Ratio	95% Confidence Interval	*p*-Value
Serum 25-hydroxyvitamin D concentrations, ng/mL		0.97	0.95–0.99	0.048
Sex	Male	Reference		
	Female	35.0	13.89–88.08	<0.001
Father’s education level	≥secondary	Reference		
	<secondary	2.81	1.39–5.70	0.004
Mother’s occupation	Housewife	Reference		
	Employed	2.44	0.61–9.68	0.206
Father’s occupation	Laborer	Reference		
	Skilled worker	2.08	1.05–4.12	0.036
Cigarette smoking/snuff	No	Reference		
	Yes	0.93	0.30–2.85	0.894

## Data Availability

The data of this study are available from the corresponding author upon reasonable request due to privacy.
